# Potential Relationship between Clinical Significance and Serum Exosomal miRNAs in Patients with Multiple Myeloma

**DOI:** 10.1155/2019/1575468

**Published:** 2019-12-14

**Authors:** Zhi-yao Zhang, Yan-chen Li, Chuan-ying Geng, Hui-juan Wang, Wen-ming Chen

**Affiliations:** Department of Hematology, Beijing Chao-yang Hospital, Capital Medical University, Beijing 100020, China

## Abstract

This study evaluated the potential relationship between exosomal miRNAs and clinical symptoms in patients with multiple myeloma (MM). Forty-eight newly diagnosed myeloma patients and sixteen normal donors were enrolled in the study. The results showed that the relative expression levels of let-7c-5p, let-7d-5p, miR-140-3p, miR-185-5p, and miR-425-5p in the exosomes of MM patients were significantly lower than those of healthy controls. Furthermore, there were significant differences in the clinical characteristics of myeloma, such as kidney damage, while the expression levels of the same miRNA in exosomes and serum are not correlated. The expression of exosomal miRNA is related to the expression levels of clinical feature-related factors, such as creatinine, *β*2-microglobulin, *β*-CTX, and IL-6 in serum. Establishing this relationship could contribute to understanding the pathogenesis of MM.

## 1. Introduction

Multiple myeloma (MM) is a malignant tumor characterized by the expansion and proliferation of abnormal monoclonal plasma cells. The clinical features of MM mainly involve bone destruction, renal damage, anemia, and immune changes, which are usually attributed to the excessive production of monoclonal immunoglobulins from tumor cells [1].

miRNAs are a small class of noncoding RNA molecules that inactivate genes by degrading or translating specific proteins [2]. miRNAs are involved in a variety of health issues and abnormal metabolic reactions such as cell proliferation, tumor invasion, angiogenesis, apoptosis, and cancer immunity. Peripheral miRNAs have been extensively studied using a noninvasive method to predict a variety of malignancies due to the simplicity and reproducibility of sample collection. miRNA has been reported to perform well in the diagnosis and treatment of multiple myeloma since it is a potential predictor of MM patients' sensitivity and resistance to myeloma-targeted drugs [3].

Exosomes are small membrane vesicle structures derived from various body fluids, such as plasma and urine, which contain a variety of information factors, including mRNA and miRNA, and small molecular proteins. Exosomal miRNA is relatively stable since exosomes can block RNase degradation due to the vesicle structure to protect genetic factors inside [4, 5]. Studies have shown that exosomal miRNA can be used as a diagnostic factor for a variety of malignancies including MM [6, 7]. The role of exosomal miRNAs in MM has also become a highly researched topic.

The aim of this study was to explore the differential expression of serum exosomal miRNAs in multiple myeloma (MM) patients and healthy control (HC) individuals as well as their relevance to the clinical symptoms of MM to explore its underlying mechanisms. In addition, serum miRNAs, which may reflect different aspects from exosome, may also suggest microenvironmental changes. Considering the complexity and high cost of the extraction of exosomal miRNA, whether the detection of exosome miRNA could be replaced by serum miRNA is also a problem that needs to be explored in this study.

## 2. Materials and Methods

### 2.1. Patient Samples

Forty-eight newly diagnosed myeloma patients from Beijing Chao-yang Hospital from April 2018 to October 2018 were enrolled in the study. All patients met the diagnostic criteria for multiple myeloma by IMWG. Myeloma patients and healthy donors were not exposed to any known cytotoxic treatment at the time of sample collection. Both myeloma patients and healthy donors signed informed consent forms.

### 2.2. Sample Preparation

Peripheral blood (2 ml) from each subject was collected in EDTA-treated tubes and stored at 4°C for no more than 1 hour. Peripheral blood was centrifuged at 3000 rpm for 15 min and then stored at −80°C until use.

### 2.3. Isolation of Exosomes and Extraction/Purification of Exosomal RNAs

The extraction and identification of exosomes and the extraction of RNA from exosomes and serum have been detailed in our previous studies [8]. ExoQuick Exosome Precipitation Solution (System Biosciences, Mountain View, CA, USA) was used to separate exosomes from serum. Twenty-five fmol synthetic *C. elegans* miRNA (Cel-miR-39, Ambion, Austin, TX, USA) was added to each sample as an internal control [9]. The miRNeasy Micro Kit (Qiagen, Hilden, Germany) was used to extract exosome samples. During the extraction and purification of RNA, the extraction step was strictly based on the protocol. The concentration and purity of RNA was detected by NanoDrop (NanoDrop Technologies, Wilmington, DE, USA).

### 2.4. Western Blotting Analysis

Separation of exosomes was identified by TS101-W in Western blot analysis. Separated exosome pellets from serum were treated with RIPA lysis buffer. The serum exosomal preparation was incubated with rabbit polyclonal anti-human TS101 IgG, followed by goat anti-rabbit horseradish peroxidase (System Biosciences).

### 2.5. Microarray Profiling

130 ng of the total RNA in each sample was enrolled in this study and hybridized for 16 h at 45°C on GeneChip following fragmentation. GeneChips were washed and stained in the Affymetrix Fluidics Station 450 and then scanned by Affymetrix® GeneChip Command Console installed on GeneChip® Scanner 3000 7G.

### 2.6. Identification of Differentially Expressed miRNAs

Data were analyzed with Robust Multichip Analysis (RMA) algorithm and values presented are log2 RMA signal intensity. *p* < 0.05 and fold change >1.5 were considered as differential expression genes. The data had been transferred to GEO (GSE124489).

### 2.7. Isolation of Serum RNA

According to the manufacturer's protocol, miRNeasy Serum/Plasma Kit (Qiagen, Hilden, Germany) was used to extract the total RNA. Twenty-five fmol of synthetic Cel-miR-39 (Ambion) was then spiked into the mixture. RNA extraction was performed following the manufacturer's protocol. NanoDrop was used to measure RNA concentration and purity.

### 2.8. Measurement of Serum Exosomal miRNA Levels and Serum Circulating miRNA Levels

Serum exosomal miRNA levels and serum circulating miRNA levels were examined by real-time quantitative PCR. Preamplification was performed after the reverse transcription of 10 ng of the total RNA with a TaqMan miRNA Reverse Transcription Kit (Applied Biosystems, Carlsbad, CA, USA) with a miRNA specific stem loop primer (TaqMan miRNA Assay Kit; Applied Biosystems). Target miRNAs were selected based on previous microarray studies (GSE124489). According to the TaqMan miRNA Assay Protocol, amplification was performed using a 7500 real-time PCR system (Applied Biosystems), and the results were analyzed using RQ Manager software (Applied Biosystems). Amplification results were tested by threshold cycle (Ct) value, and the value of each sample was calculated after the PCR was repeated twice. The spiked Cel-miR-39 was used as an internal control. The relative gene expression values of the target miRNA were normalized to Cel-miR-39 and calculated using the 2-ΔΔCT method [10, 11].

### 2.9. Enzyme-Linked Immunosorbent Assay (ELISA)

Secretion of IL-6, IL-6R, VEGF, 25-OH-VD, BAP, and *β*-CTX was assessed in peripheral blood plasma using ELISA quantification kits (IL-6 and IL-6R from Diaclone Biotechnology Ltd., France; VEGF and *β*-CTX from PHIDA Biotechnology Ltd., China; 25-OH-VD and BAP from IDS Biotechnology Ltd., UK). The assay was performed according to the protocol provided by the manufacturer.

### 2.10. Statistical Analysis

miRNA values are described as the mean ± SD. Differences between the two groups were analyzed by the Mann–Whitney *U* test, and Dunn's comparative test was used as a posttest. Spearman assessed the correlation (*r*) of miRNA expression between serum exosomes and circulating miRNAs. All data were statistically analyzed by 24.0 SPSS (SPSS Inc., Chicago, IL, USA). Statistical significance was considered positive when the *p* value was <5%.

## 3. Results

### 3.1. Selected miRNA Profiling Based on Previous Investigation and Exosomes Verified by Electron Microscope and Western Blotting

**Figure 1:**
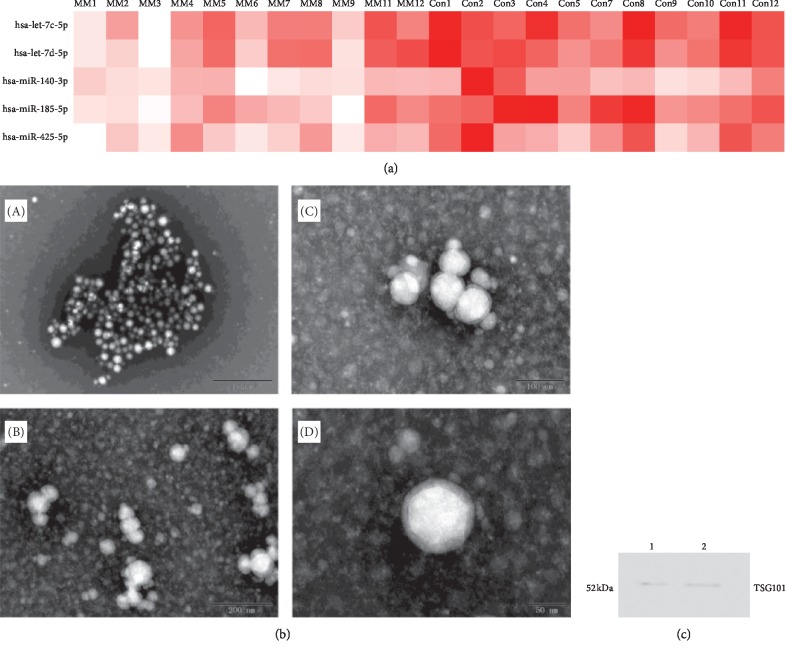
Aberrant miRNAs in the microarray and identification of serum exosomes. (a) Expression of miRNAs selected from previous exosome microarray results. (b) Exosomes of MM patients' serum purified by the kit method and verified by electron microscopy with scale bar of (A) 1 *μ*m, (B) 200 nm, (C) 100 nm, and (D) 50 nm. (c) Exosomes were verified by Western blotting. The exosomal marker TSG101 is enriched in the extraction.

miRNA profiling results, as shown in Table 1, were analyzed based on the results of the microarray, where let-7c-5p, let-7d-5p, miR-140-3p, miR-185-3p, and miR-425-5p were significantly decreased compared with those of healthy controls (Figure 1(a)). Exosomes with a diameter of approximately 50–60 nm were observed by electron microscope (Figure 1(b)). TS101 was used to identify serum exosomes. We test the expression of TSG101 in isolated exosomes derived from the patient serum (Figure 1(c)).

### 3.2. miRNA Expression in Patients with Different Clinical Features of MM

**Figure 2:**
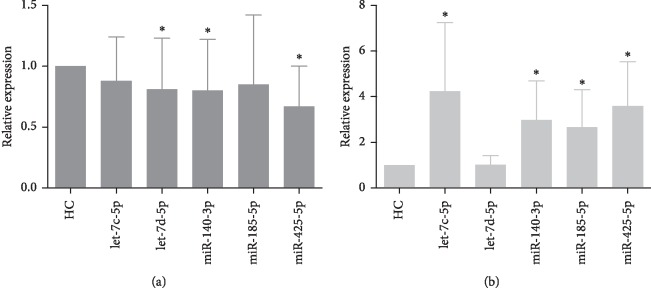
Exosomal miRNAs (a) and serum miRNAs (b) extracted from healthy control (HC) individuals and multiple myeloma (MM) patients. The expression levels of different miRNAs were measured by real-time quantitative PCR, and the relative gene expression levels were normalized based on spike-in control Cel-miR-39. The results were calculated by the 2^−ΔΔCT^ method. ^*∗*^The serum exosomal miRNA expression levels were decreased in MM patients compared with those in the HC group (*p* < 0.05). Values are expressed as the mean ± SD.

**Figure 3:**
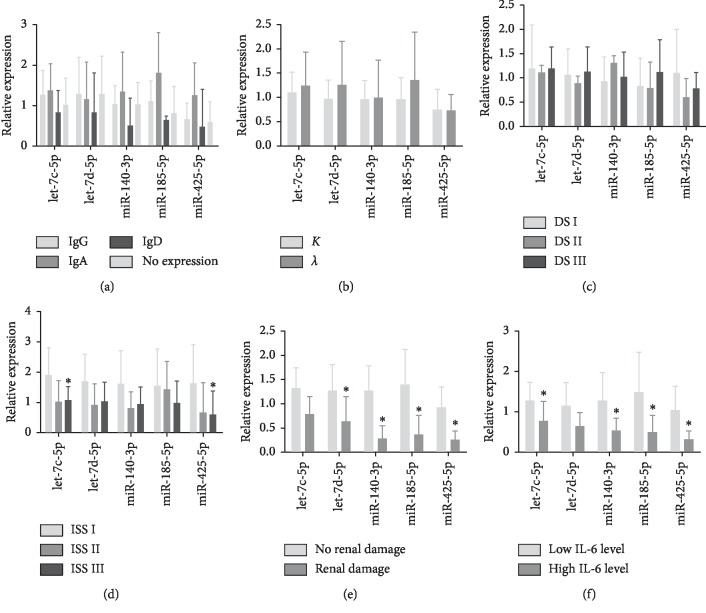
Expression of exosomal miRNAs with different clinical features. miRNA expression levels in the exosomes from serum of MM patients. Expression levels of exosomal miRNAs in (a) heavy chain style, (b) light chain style, (c) DS stage, (d) ISS stage, (e) renal damage, and (f) IL-6 level. The expression levels of different miRNAs were measured by real-time quantitative PCR, and the relative gene expression levels were normalized based on spike-in control Cel-miR-39.^*∗*^*p* < 0.05. Data represent the means ± SD.

We further analyzed the expression profile of miRNA and the clinical features of myeloma patients. First, we tested the expression levels of exosomes and serum miRNAs in 48 newly diagnosed myeloma patients. The results showed that exosomal miRNA decreased at different levels while, on the contrary, serum miRNAs expression increased (Figure 2). Among them, the expression levels of let-7d-5p, miR-140-3p, and miR-425-5p were significantly decreased in exosomes compared with those of healthy controls (*p*=0.012, *p*=0.025, and *p* < 0.001, respectively), while the expression levels of let-7c-5p, miR-140-3p, and miR-425-5p were increased in serum (*p*=0.022, *p*=0.036, and *p*=0.040, respectively). Correlation analysis showed no significant correlation between exosome and serum let-7c-5p, let-7d-5p, miR-140-3p, miR-185-5p, and miR-425-5p expression levels (*p*=0.725; *p*=0.360; *p*=0.160; *p*=0.955; *p*=0.957, respectively) (Table 2). Second, in the analysis of miRNAs with clinical symptoms, exosomes and miRNAs showed no significance in sex, age, heavy/light chain, and DS stage of the patients (Figure 3, Table 3). The expression levels of let-7d-5p and miR-425a-5p were significantly lower in ISS stage III and stage II compared with stage I. Compared with patients without kidney injury, the expressions of let-7c-5p, let-7d-5p, miR-140-3p, miR-185-5p, and miR-425-5p were decreased in patients with kidney damage. The cohort with high IL-6 levels showed increased expression of let-7c-5p, miR-140-3p, miR-185-5p, and miR-425-5p. These results indicate that the decline in exosomal miRNA expression may be associated with the development of clinical symptoms of myeloma and the progression of the disease.

### 3.3. Correlation between miRNA Expression and Different Clinical Features of MM

The results show that the expression levels of miRNAs are related to the clinical characteristics of myeloma (Table 4). Among them, miR-185-5p had positive correlation with hemoglobin, and let-7c-5p, miR-140-3p, miR-185-5p, and miR-425-5p showed negative correlation with increasing creatinine and IL-6 levels. *β*-CTX, which is an indicator reflecting the activity of osteoclasts, showed negative correlation with detected exosomal miRNAs. All detected miRNAs showed strong negative correlations in disease progression and tumor burden indicators such as *β*2-microglobulin and myeloma plasma cell load.

## 4. Discussion

MM is a type of B-cell malignancy that is currently incurable. Myeloma cells originate from cells that mediate body fluid immunity. Myeloma is mainly characterized by abnormal secretion of cytokines, abnormal activation of oncogenes, and molecular genetic abnormalities, which play an important role in the occurrence and development of myeloma diseases. In this study, we examined miRNA profiles in MM serum exosomes and calculated uniquely expressed miRNAs. The expression level of specific miRNAs was verified in patients with multiple myeloma. The expression level of miRNA in exosomes in serum was analyzed with the clinical manifestations of the disease.

The expression levels of miRNAs in exosomes and serum were observed to be inconsistent in this study, and no significant correlation was found. Sources of serum RNA include serum circulating RNA, circulating tumor cells, circulating tumor DNA, and exosomes. Exosomes can transfer small molecules from cells to cells to transmit genetic information, which can then be integrated into other cells. Serum and exosomal miRNAs represent different aspects of microenvironmental changes, respectively. Our results show that there is no significant correlation between serum miRNA and exosomal miRNA expression levels, so these two different assays cannot be substituted for each other. Furthermore, due to the presence of RNase, detection of serum miRNAs may result in unstable assays, and specific structures of exosomes may protect RNA from degradation. In conclusion, exosomal miRNAs may be candidates for improved multiple myeloma tumor markers.

Our results showed that the expression levels of let-7d-5p, miR-140-3p, and miR-425-5p in serum exosome of patients with multiple myeloma were significantly lower than those of healthy controls. This result is consistent with the results of miRNA microarrays that are considered to be tumor suppressor genes in a variety of tumor studies. Based on previous studies, the let-7 family miRNAs are lower expressed in both human cancers and stem cells [12, 13]. The let-7 family of miRNAs can regulate the cell cycle, proliferation, and apoptosis by inhibiting pluripotency promoter LIN28, targeting many of the metabolites that regulate tumorigenesis [14]. In the present study, decreased expression of the let-7 family of miRNAs in peripheral blood exosomes of myeloma patients suggests that it may play a role in the pathogenesis of myeloma. Studies have indicated that the overexpression of miR-140-3p enhanced the antitumor effect and the low miR-140-3p level is associated with osteoporosis, which could lead to an increased risk of fractures and could work as a potential biomarker candidate for osteoporosis in postmenopausal women [15]. The expression of miR-425 was revealed to be low in NPC tissues and cell lines. Resumption of miR-425 expression suppressed cell viability and invasion in nasopharyngeal carcinoma [16]. Studies have indicated that miR-185 inhibits proliferation, survival, and invasion of colorectal cancer (CRC), while upregulation of miR-185 enhances the sensitivity of CRC cells to ionizing radiation [17]. All of the above miRNAs may be present as tumor suppressor genes in a variety of tumors.

Myeloma-specific clinical symptoms mainly include kidney damage, bone marrow destruction, and bone destruction. The correlation analysis found that the above abnormally expressed miRNAs have a significant correlation with IL-6, creatinine, and osteoclast-associated factor *β*CTX expression, suggesting that abnormal miRNA may be related to not only myeloma cell growth but also the characteristics of myeloma. A certain relationship between the occurrence of clinical symptoms might exist. Interleukin 6 (IL-6) is an important growth factor in MM cells [18], and IL-6-related signaling pathway has been shown to regulate MM cell proliferation and programmed cell death in myeloma [19]. Previous studies have shown that elevated serum IL-6 levels are closely related to poor prognosis and short survival time [20]. Studies have shown that stromal cells in the microenvironment of myeloma may inhibit the expression of miRNA in myeloma cells by secreting high levels of IL-6, thereby inhibiting cell proliferation, reducing the sensitivity of myeloma cells to chemotherapeutic drugs, and participating in the occurrence of MM resistance [21–23]. There may be a complex regulatory network between miRNA and IL-6 in MM that promotes the progression of myeloma. A previous study had found that IL-6 is a direct target gene for let-7 miRNA, which was downregulated in cancer-associated MSCs. Downregulation of let-7 by cancer-associated MSCs upregulates IL-6 expression [23, 24]. Our correlation analysis suggests that dysregulation of miRNA may affect the level of IL-6 and contribute to the pathogenesis and progression of MM.

In addition, the expression level of exosomal miRNA is negatively correlated with creatinine level, bone marrow plasma cell load, and expression levels of serum factors such as *β*2-microglobulin and *β*-CTX (*β*-type I collagen carboxy-terminal peptide) in myeloma patients, suggesting that the expression level of miRNA in multiple myeloma exosomes is correlated with clinical manifestations of myeloma. It is worth mentioning that *β*2-microglobulin is associated with both tumor load and kidney injury in myeloma. In this study, there was no obvious significant correlation between kidney injury and *β*2-microglobulin levels (*r* = 0.153, *p*=0.30). Therefore, we believe that the significant differences in *β*2-microglobulin in this study are mainly related to tumor load in myeloma patients. Establishing this relationship helps to understand the pathogenesis of MM, and further study is needed.

Our studies indicate that changes in MM exosomal miRNA expression levels are closely related to the development and progression of myeloma disease. Due to the limited number of subjects involved in this study, further research is needed to elucidate the role of exosomal miRNA in the MM signaling pathway so that it may be an effective biomarker for disease prediction in MM patients.

## Figures and Tables

**Table 1:** Altered microRNA expression in serum exosomes of multiple myeloma.

MicroRNA	*p* value	Regulation	FC
hsa-miR-4741	0.0011101	Down	−1.335895
hsa-miR-185-5p	0.0016654	Down	−2.822401
hsa-mir-6090	0.0027411	Down	−1.383277
hsa-let-7d-5p	0.0045661	Down	−2.445867
hsa-miR-4505	0.0080223	Down	−1.421201
hsa-miR-19b-3p	0.008502	Down	−1.478023
hsa-let-7c-5p	0.0101621	Down	−1.993793
hsa-miR-425-5p	0.0101974	Down	−1.464822
hsa-miR-6849-5p	0.0104978	Down	−1.34703
hsa-miR-3162-5p	0.011158	Down	−1.300645
hsa-miR-6891-5p	0.0116116	Down	−1.452478
hsa-miR-140-3p	0.0127439	Down	−1.359699
hsa-miR-4632-5p	0.0131308	Down	−1.455522
hsa-miR-103a-3p	0.0146007	Down	−1.997548
hsa-let-7i-5p	0.0149937	Down	−1.387171
hsa-miR-6749-5p	0.0166741	Down	−1.559853
hsa-miR-885-3p	0.0177255	Down	−1.3652
hsa-miR-6750-5p	0.0198816	Down	−1.54725
hsa-miR-937-5p	0.0199458	Down	−1.450515
hsa-miR-20a-5p	0.020742	Down	−1.65547
hsa-miR-6824-5p	0.0208769	Down	−1.489712
hsa-miR-361-5p	0.0210404	Down	−1.361168
hsa-miR-1233-5p	0.0248246	Down	−1.36092
hsa-miR-1909-3p	0.0279793	Down	−1.42102
hsa-miR-451a	0.0282091	Down	−1.641756
hsa-miR-328-5p	0.0292033	Down	−1.645904
hsa-miR-6775-5p	0.0306503	Down	−1.51946
hsa-miR-3141	0.0314553	Down	−1.403586
hsa-miR-1343-5p	0.0317415	Down	−1.419553
hsa-miR-503-5p	0.0342952	Down	−1.352801
hsa-miR-4459	0.0346482	Down	−1.356296
hsa-miR-6743-5p	0.0347592	Down	−1.449548
hsa-miR-4429	0.036453	Down	−1.416416
hsa-miR-4701-3p	0.0387719	Down	−1.390077
hsa-miR-4706	0.0404707	Down	−1.330192
hsa-miR-1246	0.0430674	Down	−2.145079
hsa-miR-4433-3p	0.0464502	Down	−1.303152
hsa-mir-4281	0.0481501	Down	−1.309318
hsa-miR-5189-3p	0.0019957	Up	3.319253
hsa-miR-4532	0.003398	Up	4.3523215
hsa-miR-1273g-3p	0.0095161	Up	2.0281869
hsa-miR-2115-5p	0.0215373	Up	2.0663002
hsa-miR-3665	0.0420994	Up	1.7028116

**Table 2:** Correlation of the expression level between exosomal and serum miRNAs.

miRNAs	*r*	*p* value
Serum and exosomal let-7c-5p	0.066	0.725
Serum and exosomal let-7d-5p	−0.153	0.360
Serum and exosomal miR-140-3p	0.239	0.160
Serum and exosomal miR-185-5p	−0.009	0.955
Serum and exosomal miR-425-5p	−0.009	0.957

“*r*” indicates the correlation coefficient between serum exosomal and circulating microRNAs.

**Table 3:** Clinical features of multiple myeloma patients and the expression of exosomal miRNAs in different groups.

Characteristics	*N* (%)	let-7c-5p	*p*	let-7d-5p	*p*	miR-140-3p	*p*	miR-185-5p	*p*	miR-425-5p	*p*
	(*n* = 48)	Mean ± SD		Mean ± SD		Mean ± SD		Mean ± SD		Mean ± SD	
*Age*			**0.487**		**0.616**		**0.756**		**0.321**		**0.435**
≥65	15 (31%)	1.32 ± 0.91		1.22 ± 1.11		1.09 ± 1.04		1.56 ± 2.01		1.94 ± 1.11	
<65	33 (69%)	1.12 ± 0.84		1.06 ± 0.99		1.08 ± 0.96		0.98 ± 0.91		1.69 ± 0.58	

*Sex*			**0.456**		**0.605**		**0.169**		**0.956**		**0.404**
Male	22 (46%)	1.32 ± 1.01		1.23 ± 1.22		0.86 ± 0.91		1.34 ± 1.73		0.67 ± 0.67	
Female	26 (54%)	1.06 ± 0.71		1.00 ± 0.82		1.15 ± 1.02		0.99 ± 0.92		0.86 ± 0.86	

*Heavy chain*			**0.479**		**0.505**		**0.266**		**0.501**		**0.101**
No expression	15 (31%)	1.02 ± 0.62		1.29 ± 0.67		1.03 ± 0.66		0.81 ± 0.72		0.59 ± 0.67	
IgG	16 (34%)	1.27 ± 0.87		1.29 ± 1.23		1.04 ± 0.68		1.11 ± 0.88		0.66 ± 0.39	
IgA	13 (27%)	1.37 ± 0.95		1.16 ± 1.12		1.34 ± 1.38		1.81 ± 2.21		1.26 ± 1.14	
IgD	4 (8%)	0.83 ± 0.48		0.83 ± 0.47		0.50 ± 0.45		0.64 ± 0.86		0.46 ± 0.64	

*Light chain*			**0.805**		**0.846**		**0.366**		**0.568**		**0.595**
Nonsecretion	1 (2%)	—		—		—		—		—	
*Κ*	27 (56%)		1.10 ± 0.72		0.97 ± 0.68		0.96 ± 0.68		0.96 ± 0.75		0.75 ± 0.75
*Λ*	20 (42%)	1.24 ± 1.02		1.26 ± 0.1.36		0.99 ± 1.21		1.36 ± 0.1.81		0.73 ± 0.74	

*D-S stage*			**0.840**		**0.852**		**0.444**		**0.940**		**0.882**
I	7 (15%)	1.19 ± 0.79		1.06 ± 0.74		0.93 ± 0.63		0.83 ± 0.67		1.10 ± 1.30	
II	2 (4%)	1.11 ± 0.07		0.89 ± 0.03		1.31 ± 0.00		0.79 ± 0.25		0.60 ± 0.19	
III	39 (81%)	1.19 ± 0.90		1.13 ± 1.09		1.02 ± 1.05		1.12 ± 1.45		0.78 ± 0.68	

*ISS stage*			**0.047** ^*∗*^		**0.055**		**0.083**		**0.072**		**0.027** ^*∗*^
I	7 (15%)	1.91 ± 0.92		1.69 ± 0.79		1.61 ± 1.00		1.55 ± 1.02		1.63 ± 1.25	
II	11 (23%)	1.03 ± 0.76		0.92 ± 0.75		0.81 ± 0.58		1.43 ± 0.98		0.67 ± 0.47	
III	30 (62%)	1.07 ± 0.82		1.04 ± 1.12		0.94 ± 1.05		0.98 ± 1.43		0.60 ± 0.60	

*Renal damage*			**0.066**		**0.011** ^*∗*^		**0.002** ^*∗*^		**0.026** ^*∗*^		**0.010** ^*∗*^
Yes	12 (25%)	0.79 ± 0.74		0.64 ± 0.49		0.28 ± 0.27		0.37 ± 0.42		0.26 ± 0.19	
No	36 (75%)	1.32 ± 0.86		1.27 ± 0.1.10		1.27 ± 1.00		1.40 ± 1.45		0.93 ± 0.84	

ISS, International Staging System; ^*∗*^*p* < 0.05.

**Table 4:** Correlation between the expression of miRNA and different clinical characteristics.

Characteristics	let-7c-5p		let-7d-5p		miR-140-3p		miR-185-5p		miR-425-5p	
	*r*	*p*	*r*	*p*	*r*	*p*	*r*	*p*	*r*	*p*
Hemoglobin	0.107	*0.469*	0.222	*0.129*	0.306^*∗*^	*0.034*	0.325^*∗*^	*0.027*	0.279	*0.058*
Neutrophil	0.038	*0.802*	0.020	*0.892*	−0.077	*0.609*	0.044	*0.773*	0.000	*0.998*
Platelet	0.115	*0.440*	0.106	*0.477*	0.256	*0.083*	0.259	*0.086*	0.296^*∗*^	*0.046*
Creatinine	−0.309^*∗*^	*0.033*	−0.227	*0.120*	−0.527^*∗∗*^	*0.000*	−0.500^*∗∗*^	*0.000*	−0.524^*∗∗*^	*0.000*
Ca	−0.140	*0.341*	−0.076	*0.608*	0.034	*0.821*	−0.013	*0.930*	0.076	*0.609*
*β*2-Microglobulin	−0.311^*∗*^	*0.031*	−0.412^*∗∗*^	*0.004*	−0.437^*∗∗*^	*0.002*	−0.483^*∗∗*^	*0.001*	−0.453	*0.001*
ALP	−0.097	*0.513*	−0.088	*0.551*	0.096	*0.515*	0.078	*0.606*	0.044	*0.771*
LDH	−0.032	*0.828*	0.017	*0.907*	−0.201	*0.172*	−0.140	*0.354*	−0.197	*0.185*
VEGF	−0.001	*0.996*	−0.147	*0.377*	−0.106	*0.526*	−0.152	*0.370*	−0.105	*0.529*
IL-6	−0.426^*∗∗*^	*0.009*	−0.244	*0.146*	−0.393^*∗*^	*0.016*	−0.420^*∗*^	*0.011*	−0.518^*∗∗*^	*0.001*
IL-6R	−0.206	*0.215*	−0.091	*0.585*	−0.038	*0.823*	−0.026	*0.877*	−0.096	*0.568*
TRAP5B	−0.070	*0.681*	0.075	*0.661*	−0.057	*0.738*	0.038	*0.828*	0.022	*0.897*
*β*-CTX	−0.326^*∗*^	*0.047*	−0.334^*∗*^	*0.044*	−0.346^*∗*^	*0.036*	−0.349^*∗*^	*0.037*	−0.415^*∗*^	*0.011*
25-OH-VD	0.124	*0.465*	0.128	*0.450*	0.177	*0.296*	0.248	*0.144*	0.237	*0.158*
BAP	0.010	*0.954*	0.075	*0.660*	−0.058	*0.731*	0.058	*0.737*	0.090	*0.596*
BM PC (%)	−0.311^*∗*^	*0.031*	−0.412^*∗∗*^	*0.004*	−0.437^*∗∗*^	*0.002*	−0.483^*∗∗*^	*0.001*	−0.453^*∗∗*^	*0.001*

^*∗*^ *p* < 0.05; ^*∗∗*^*p* < 0.01; *r*, correlation; IL-6, interleukin-6; ALP, alkaline phosphatase; LDH, lactate dehydrogenase; VEGF, vascular endothelial growth factor; IL-6R, interleukin-6 receptor; TRAP5B, tartrate-resistant acid phosphatase; *β*-CTX, *Β*-type I collagen carboxy-terminal peptide; 25-OH-VD, 25-hydroxyvitamin D; BAP, bone alkaline phosphatase; BM PC, bone marrow plasma cells.

## Data Availability

The data used to support the findings of this study are available from the corresponding author upon request.
